# Idiopathic Bilateral Extraocular Myositis in a Subject With Poorly Controlled Type 2 Diabetes Mellitus: Case Report

**DOI:** 10.3389/fmed.2021.700307

**Published:** 2021-09-30

**Authors:** Fuminori Tatsumi, Yoshiro Fushimi, Junpei Sanada, Masashi Shimoda, Kenji Kohara, Tomohiko Kimura, Atsushi Obata, Shuhei Nakanishi, Tomoatsu Mune, Kohei Kaku, Hideaki Kaneto

**Affiliations:** ^1^Department of Diabetes, Endocrinology and Metabolism, Kawasaki Medical School, Kurashiki, Japan; ^2^Kawasaki Medical School General Medical Center, Kurashiki, Japan

**Keywords:** idiopathic bilateral extraocular myositis, extraocular muscle paralysis, type 2 diabetes mellitus, diabetic neuropathy, a rare case

## Abstract

**Background:** Extraocular myositis is characterized by acute onset of orbital pain, extraocular muscle swelling, absence of thyroid disease, and effectiveness of steroid therapy. While oculomotor nerve paralysis is often observed in subjects with diabetes mellitus, extraocular muscle paralysis is very rare among various diabetic mononeuropathies. In addition, while most diabetic mononeuropathies are observed as sporadic and/or unilateral neuropathy, bilateral mononeuropathy is also very rare.

**Case presentation:** A 58-year-old male visited our institution due to diplopia. He was diagnosed as type 2 diabetes mellitus about 10 years before and treated with oral diabetes agents. To examine the cause of his symptom, he was hospitalized in our institution. Slight ptosis was observed, and failure of adduction was observed in the right eye. Glycemic control was poor; HbA1c was 9.3%. Liver, renal, and thyroid function were within normal range. Immunoglobulin (Ig) G was slightly high, but IgA, IgM, and IgG4 were within normal range. Various antibodies were all negative. Angiotensin-converting enzyme level was within normal range. There were no abnormalities in brain magnetic resonance imaging (MRI). After admission, to alleviate glucose toxicity, we started insulin therapy. On day 17, adduction failure of the left eye was observed in addition to the right eye. Vertical movement was also impaired in both eyes. Slight ptosis was observed in both eyes, and the right eye was completely close. In orbital MRI, some high signal was detected in both extraocular muscles. We performed steroid pulse therapy twice. About 4 months later, ptosis and vertical and horizontal movements in both eyes were almost completely recovered. Finally, we diagnosed him as idiopathic bilateral extraocular myositis.

**Conclusions:** We should bear in mind the possibility of idiopathic bilateral extraocular myositis especially in subjects with poor glycemic control, although its incident rate is extremely rare.

## Introduction

Extraocular myositis is characterized by acute onset, orbital pain, extraocular muscle swelling, absence of thyroid disease, and effectiveness of steroid therapy (1). While it is well-known that oculomotor nerve paralysis is often observed in subjects with diabetes mellitus, extraocular muscle paralysis is very rare among various diabetic mononeuropathies. It was reported that the percentage of extraocular paralysis was as small as about 1 % in diabetic mononeuropathies (2, 3). Furthermore, while most of diabetic mononeuropathies are observed as sporadic and/or unilateral neuropathy, bilateral mononeuropathy is very rare (4). Here we show a subject with type 2 diabetes mellitus who had idiopathic bilateral extraocular myositis.

## Case Report

**Figure 1:**
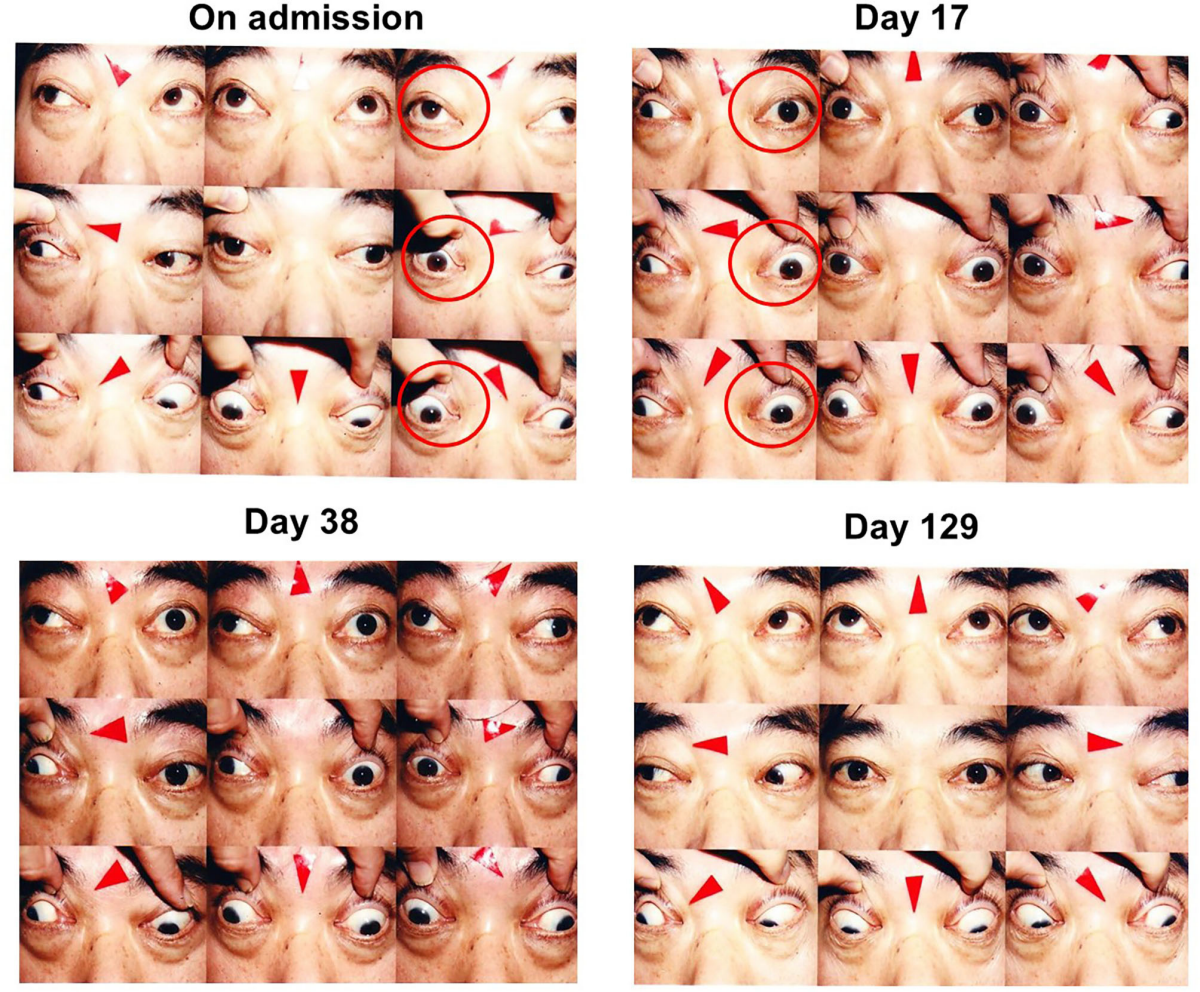
On admission, slight ptosis was observed, and adduction failure of the right eye was observed (**left**, upper panels; red circles). On day 17, adduction failure of the left eye was observed in addition to that of the right eye. Vertical movement was also impaired in both eyes. Slight ptosis was observed in both eyes, and the right eye was completely close (**right**, upper panels; red circles). On day 38 after steroid therapy, ptosis and vertical movements in both eyes were recovered but horizontal movements of both eyes were not altered (**left**, lower panels). On day 129, ptosis and vertical movements in both eyes were completely normalized and horizontal movements of both eyes were almost completely recovered (**right**, lower panels).

A 58-year-old male visited our institution due to diplopia. He was diagnosed as type 2 diabetes mellitus about 10 years before and treated with oral diabetes agents (1.5 mg of glimepiride, 15 mg of pioglitazone, 500 mg of metformin). To examine the cause of his symptom, he was hospitalized in our institution. His height and body weight were 165.3 cm and 57.2 kg. Blood pressure and heart rate were 112/68 mmHg and 78 /min. Slight ptosis was observed, and failure of adduction was observed in the right eye (Figure 1). Glycemic control was poor; HbA1c and plasma glucose levels were 9.3% and 223 mg/dL. Liver, renal and thyroid function were within normal range. Immunoglobulin (Ig) G was slightly high (2,117 mg/dL), but IgA, IgM, and IgG4 were within normal range (IgA, 282.5 mg/dL; IgM, 222.3 mg/dL; IgG4, 23.4 mg/dL). Various antibodies were all negative: thyrotropin receptor antibody (TRAb), <1.0 U/L; thyroid-stimulating hormone antibody (TSAb), 102%; antinuclear antibody, 18.6-fold; rheumatoid factor, <15 U/mL, anti-acetylcholine receptor antibody (anti-Ach-R Ab), ≤0.2; myeloperoxidase-anti-neutrophil cytoplasmic antibody (MPO-ANCA), <1.0 U/mL; proteinase 3-anti-neutrophil cytopkasmic antibody (PR3-ANCA), 2.8 U/mL; human immunodeficiency virus (HIV) antibody, negative; human T-cell leukemia virus type 1 (HTLV-1) antibody, negative; cytomegalovirus (CMV) antibody, negative; QuantiFERON, negative; treponema pallidum hemagglutination (TPHA), negative. Angiotensin-converting enzyme (ACE) level was within normal range (12.6 U/L). Diabetic retinopathy was not observed, and diabetic nephropathy and neuropathy were mild: albuminuria, 34.2 mg/g.Cre; vibration sense, 12/11 s in upper limbs, 8/9 s in lower limbs. There were no abnormalities in brain magnetic resonance imaging (MRI). Carotid average and max intima-media thickening was within normal range (right/left: 0.63/0.66 mm, 0.75/0.82 mm). After admission, in order to alleviate glucose toxicity, we started insulin therapy with glulisine and glargine U300 and adjusted insulin dose. Approximately 2 months later, HbA1c was decreased to 8.3%.

**Figure 2:**
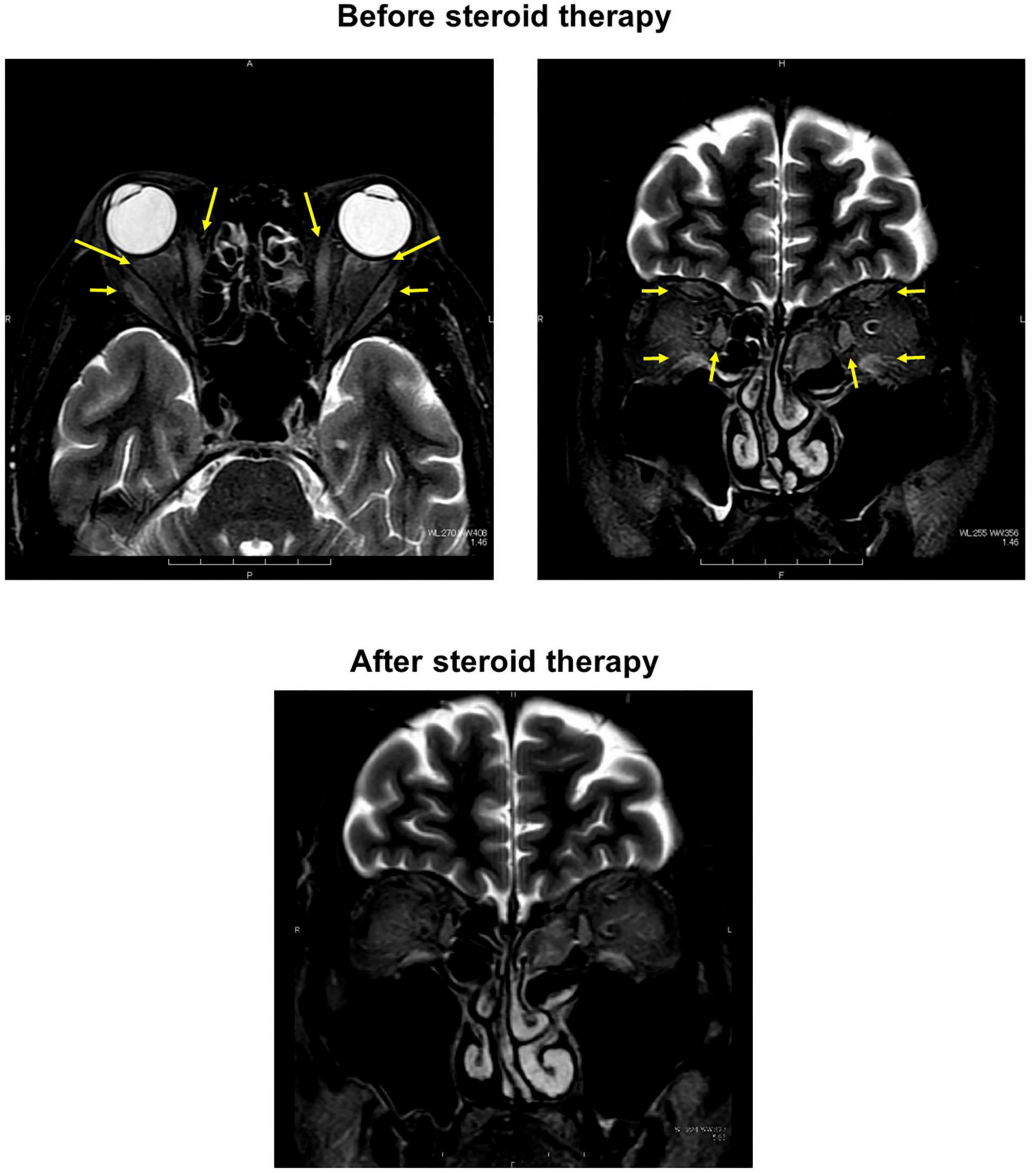
**(Upper)** In orbital magnetic resonance imaging before starting treatment, some high signal was detected in both extraocular muscles, indicating that inflammation extended into the extraocular muscles in this subject (both panels; yellow arrows). **(Lower)** After the steroid pulse therapy, signal intensity in extraocular muscle tended to be weakened, and orbital fat mass was moderately reduced.

On day 17, adduction failure of the left eye was observed in addition to the right eye. Vertical movement was also impaired in both eyes. Slight ptosis was observed in both eyes, and the right eye was completely close (Figure 1). In orbital MRI, some high signal was detected in both extraocular muscles, indicating that inflammation extended into the extraocular muscles in this subject (Figure 2). ESR was high as follows: 8 mm (30 min), 43 mm (60 min), and 80 mm (120 min), although other inflammatory markers were within normal range: WBC, 5290 /μL; CRP, 0.04 mg/dL. On day 21, we started steroid pulse therapy (methylprednisolone sodium succinate 1,000 mg/day × 3 days). Two weeks later, we repeated the same steroid therapy. After the steroid pulse therapy, signal intensity in extraocular muscle tended to be weakened, and orbital fat mass was moderately reduced (Figure 2). Since subjective symptoms were substantially reduced after then, we did not perform an additional MRI. It is noted here that poor glycemic control was brought about as a side effect of steroid therapy in this subject. Indeed, glycemic control became quite poor even with basal-bolus insulin therapy after steroid pulse therapy, although it was pretty well before the therapy. On day 38, ptosis and vertical movements in both eyes were recovered but horizontal movements of both eyes were not altered (Figure 1). On day 129, ptosis and vertical and horizontal movements in both eyes were almost completely recovered (Figure 1).

We ruled out various disorders which could bring about extraocular myositis such as autoimmune or inflammatory diseases (Graves' disease, sarcoidosis, and systemic lupus erythematosus), malignancy and cerebrovascular lesions. Finally, we diagnosed him as idiopathic bilateral extraocular myositis.

## Discussion

Here, we showed a case with idiopathic bilateral extraocular myositis in a subject who had type 2 diabetes mellitus with poor glycemic control but did not have any other overt diabetic complications. While it is well-known that oculomotor nerve paralysis is often observed in subjects with diabetes mellitus, extraocular muscle paralysis is very rare among various diabetic mononeuropathies (2, 3). Furthermore, while most of diabetic mononeuropathies are observed as sporadic and/or unilateral neuropathy, bilateral mononeuropathy is very rare (4). At first, we thought the possibility that this subject had diabetic mononeuropathy. However, since extraocular myositis was suspected in orbital MRI, we thought that this subject did not suffer from diabetic mononeuropathy, and after searching for another cause, we finally diagnosed this subject as idiopathic bilateral extraocular myositis. To the best of our knowledge, the incidence rate of bilateral extraocular myositis is extremely low.

It has been reported that extraocular myositis is induced by the presence of a variety of autoimmune and infectious diseases (5, 6). In this subject, IgG was slightly high, but IgA, IgM, and IgG4 were within normal range. Various antibodies (TRAb, TSAb, antinuclear antibody, rheumatoid factor, anti-Ach-R antibody, MPO-ANCA, PR3-ANCA, HIV antibody, HTLV-1 antibody, CMV antibody, QuantiFERON, TPHA) were all negative. ACE level was within normal range. We ruled out various disorders such as autoimmune or inflammatory diseases, and we finally diagnosed him as idiopathic bilateral extraocular myositis.

Idiopathic extraocular myositis is characterized by acute or subacute onset of orbital pain, extraocular muscle swelling, absence of thyroid disease and effectiveness of immunosuppressive treatment (7). However, this subject did not have any orbital pain. In this point, extraocular myositis in this subject is different from typical extraocular myositis. It has been reported that when inflammation extends into the extraocular muscles, ocular motility disorder is often brought about (8). As shown in brain MRI, inflammation extended into the extraocular muscles in this subject, which probably brought about ocular motility disorder.

It is known that steroid therapy shows favorable effects on extraocular myositis and that when we use steroid at the early stage of this disorder, sequelae are less likely to remain. Also, it has been shown that delayed start of steroid therapy leads to recurrence of extraocular myositis, necessity of long-term steroid therapy and/or additional radiation therapy (9–13). In this subject, short-term steroid pulse therapy exerted favorable effects and did not need additional radiation therapy probably because we started steroid therapy at an early stage of extraocular myositis. This case report strengthens the importance that we should start steroid therapy at an early stage of extraocular myositis.

Steroid therapy is usually effective for ptosis and diplopia due to Myashenia gravis. Although we did not measure MuSK, we thought that this subject did not have Myashenia gravis due to the following reasons. First, while it is known that subjective symptoms are obvious in the afternoon in subjects with eye muscle Myashenia gravis, there was no daily variation in subjective symptoms in this subject. Second, while in general exophthalmos is not observed in subjects with Myashenia gravis, this subject showed exophthalmos.

Taken together, we should bear in mind the possibility of idiopathic bilateral extraocular myositis in subjects with poorly controlled diabetes mellitus even when they do not have any other overt diabetic complications.

## Data Availability Statement

The raw data supporting the conclusions of this article will be made available by the authors, without undue reservation.

## Ethics Statement

Written informed consent was obtained from the individual for the publication of any potentially identifiable images or data included in this article.

## Author Contributions

FT and HK researched data and wrote the manuscript. YF, JS, MS, KKo, TK, AO, SN, TM, and KKa contributed to discussion. All authors contributed to the article and approved the submitted version.

## Conflict of Interest

The authors declare that the research was conducted in the absence of any commercial or financial relationships that could be construed as a potential conflict of interest.

## Publisher's Note

All claims expressed in this article are solely those of the authors and do not necessarily represent those of their affiliated organizations, or those of the publisher, the editors and the reviewers. Any product that may be evaluated in this article, or claim that may be made by its manufacturer, is not guaranteed or endorsed by the publisher.
